# Efficacy of Mulligan joint mobilizations and trunk stabilization exercises versus isometric knee strengthening in the management of knee osteoarthritis: a randomized controlled trial

**DOI:** 10.1186/s13102-024-00893-7

**Published:** 2024-05-07

**Authors:** Shaikh Nabi Bukhsh Nazir, Farooq Azam Rathore

**Affiliations:** 1 Institute of Physical Medical & Rehabilitation, Dow University of Health Sciences, Karachi, Pakistan; 2 Armed Forces Institute of Rehabilitation Medicine (AFIRM), Rawalpindi, Pakistan

**Keywords:** Exercise therapies, Isometric contraction, Kinesiotaping, Knee joint, Manual therapy, Osteoarthritis, Pakistan

## Abstract

**Background:**

Knee osteoarthritis (KOA) progression is often influenced by biomechanical factors. Biomechanical interventions, such as Trunk stabilization exercise (TSE) and Mulligan joint mobilization (MWM), may offer relief from KOA symptoms and potentially slow disease progression. However, the comparative efficacy of these therapies remains uncertain. This study aimed to compare the efficacy of TSE, Mulligan joint mobilization, and isometric knee strengthening (KSE) on disability, pain severity, and aerobic exercise capacity in patients with KOA.

**Methodology:**

A randomized controlled trial (RCT) with three intervention groups was conducted between September 2020 to February 2021. The study enrolled adults aged between 40 and 60 years with a confirmed KOA diagnosis recruited from the physical therapy clinic of the Sindh Institute of Physical Medicine and Rehabilitation, Pakistan. Participants were randomly assigned to receive 24 sessions of either TSE, MWM, or KSE. The knee’s functionality was assessed using the Knee Injury and Osteoarthritis Outcome Score (KOOS), pain on a visual analogue scale (VAS), and two objective functional tests—the 6-minute walk test (6MWT) and the 11-stair climb test (SCT). These assessments were conducted at baseline, the third week, and the sixth week. Changes in outcome measures were analyzed using a mixed-design ANOVA with Bonferroni post-hoc analysis, with statistical significance set at a p-value < 0.05.

**Result:**

Of the 60 participants, 22 (36.7%) were females, and 38 (63.3%) were males. Within-group analysis revealed a significant improvement in all outcome measures at the third week (*p* < 0.05) and sixth week (*p* < 0.05). Notably, the TSE group exhibited a greater reduction in mean difference (M.D) in VAS scores than the MWM and KSE groups across various measures in the third week. At rest, during stair ascent, and descent, the TSE group showed significant improvements in VAS scores: MWM (-2.05; -1.94; -1.94), TSE (-2.38; -2.5; -2.5), KSE (-1.05; -0.63; -0.63). Additionally, during sub-maximal exercise capacity assessment, the TSE group showed greater improvement (MWM 12.89; TSE 22.68; KSE 7.89), as well as in Knee Injury and Osteoarthritis Outcome Score for activities of daily living (KOOS-ADL) (MWM 20.84; TSE 28.84; KSE 12.68), and KOOS-pain (MWM 24.84; TSE 27.77; KSE 5.77) at the third-week assessment (*p* < 0.05). The TSE group demonstrated significant improvements (*p* < 0.05) across various measures in the sixth week. Specifically, improvements were observed in VAS scores at rest (MWM − 4.15; TSE − 4.42; KSE − 3.78), during stair ascent (MWM − 3.89; TSE − 4.88; KSE − 3.56) and descent (MWM − 3.78; TSE − 4.05; KSE − 2.94). Furthermore, significant improvements were noted in the stair climb test (MWM − 7.05; TSE − 7.16; KSE − 4.21), 6-minute walk test (6MWT) (MWM 22.42; TSE 37.6; KSE 13.84), KOOS-pain (MWM 41.47; TSE 49.11; KSE 28.73), and KOOS-ADL (MWM 40.31; TSE 50.57; KSE 26.05).

**Conclusion:**

In this study in patients with KOA, TSE had greater efficacy compared to MWM and KSE in enhancing functional levels, reducing pain, improving sub-maximal exercise capacity, and performance on the stair climb test. Importantly, mean scores between the groups, particularly in the TSE group, reached the minimally important level, particularly in key areas such as pain, functional levels, sub-maximal exercise capacity, and stair climb performance. Clinicians should consider the significant pain reduction, improved functionality, and enhanced exercise capacity demonstrated by TSE, indicating its potential as a valuable therapeutic choice for individuals with KOA.

**Trial no:**

ClinicalTrials.gov = NCT04099017 23/9/2019.

**Supplementary Information:**

The online version contains supplementary material available at 10.1186/s13102-024-00893-7.

## Background

Knee osteoarthritis (KOA) is a progressive degenerative joint disease characterized by symptoms such as knee stiffness, swelling, and loss of function. The symptoms tend to worsen over time, resulting in decreased walking speed and difficulties in tasks like climbing stairs [1, 2].

The etiology of KOA involves complex biomechanical factors, including altered joint alignment and muscle imbalances, exacerbating the degenerative process and contributing to symptom severity [3]. 

Management strategies for KOA often involve biomechanical corrections including manual treatment techniques, exercise, orthosis, and taping [4]. Specifically, taping or knee strengthening exercises in combination with manual therapy, when supervised by a qualified physiotherapist, are recommended for patients with KOA [5]. However, there remains a lack of clear guidelines regarding the type and dosage of exercises in KOA, despite the strong endorsement of knee-strengthening exercises in recent guidelines [6]. The potential benefits of twenty-four sessions of supervised exercise appear to provide the most beneficial effect [7]. However, this effect has not been explored within the context of Mulligan joint mobilization and trunk stabilization exercises.

Brian Mulligan introduced the concept of joint mobilization involving simultaneous glides and active movement, known as mobilization with movement (MWM) [8]. Biomechanically, MWM may address joint arthrokinematics by correcting positional faults, potentially restoring the normal kinematics of the osteoarthritic knee and resulting in immediate pain relief.^3–4,8−9^ However, taping with MWM is believed to help maintain the potential effects of the previous mobilization and may contribute to greater pain reduction [10]. Despite this hypothesis, there is limited research supporting the combined effects of MWM, exercise, and taping.

The body’s center of gravity location in the human body is not only influenced by the control of the lumbopelvic complex but also reflects the direction of the ground force vector, affecting knee load distribution.^11^ Individuals with KOA often exhibit increased trunk asymmetry during daily activities, contributing to increased knee pain and disability, along with quadriceps strength imbalances [12, 13]. Trunk stability exercises (TSE) can be used to control the movement of the lumbopelvic complex and lower limb alignment [14]. In support of this approach, Hernandez et al. reported that the combination of trunk stability and knee strengthening resulted in a short-term pain reduction [15].

To the best of our knowledge and online literature search, no study has comprehensively compared the effects of MWM and TSE versus isometric knee strengthening exercises in managing KOA. The aim of this study was to compare the outcomes of three treatment groups (TSE, MWM, and isometric knee strengthening exercise) to determine which treatment was most effective at improving sub-maximal exercise capacity, reducing disability, and relieving pain in patients with KOA. Given the current knowledge and the lack of comprehensive studies comparing these interventions, our null hypothesis is that there is no statistically significant difference between the three treatment groups (MWM, TSE, and isometric knee strengthening exercises) for KOA in terms of pain, disability, and sub-maximal exercise capacity. This hypothesis is based on the understanding that each intervention targets different aspects of biomechanical adaptation, yet their overall efficacy in addressing the specified outcomes may not significantly differ. Our motivation for this hypothesis is to establish evidence-based guidance for clinicians and physiotherapists in selecting the most suitable intervention for KOA, considering factors such as effectiveness, patient comfort, and long-term outcomes.

## Materials and methods

### Study design and participants

A three-arm randomized controlled trial (RCT) was conducted from September 2020 to February 2021 at the physical therapy clinic of the Sindh Institute of Physical Medicine and Rehabilitation (SIPMR), Sindh, Pakistan. Patients were recruited following referrals by physiatrists, orthopedic surgeons, and general physicians. The study adheres to CONSORT statement guidelines [16]. Eligible participants were adults aged 40 to 60 years old, diagnosed with KOA according to the American College of Rheumatology (ACR) criteria with Grade I or II osteoarthritis on Kellgren Lawrence (K/L) radiological criteria. We excluded participants with skin sensitivity or an allergy to tape, post-traumatic osteoarthritis, lower limb sensorimotor dysfunction, constitutional or non-specific symptoms (uncontrolled blood pressure, malaise, weight loss, and fever), recent intraarticular injection of any kind in the last six months, back pain, a history of spinal surgery, those using assistive devices for ambulation (sticks, walkers, and canes), patellofemoral joint arthritis, a BMI greater than 30 kg/m2, a reading on the VAS scale less than four, and severe joint deformity of the lower extremity.

### Sample size

The sample size was calculated to satisfy outcomes for both pain (VAS) and disability (KOOS). We used the Lalunpui article as a reference [17], the visual analogue scale (VAS) scores and standard deviations at the fifth week were as follows: group one had a score of 3.98 ± 0.73, group two had a score of 2.23 ± 0.73, and group three had a score of 3.12 ± 0.66. Using the G*Power 3.1.9.4 software for one-way ANOVA, it was determined that a minimum sample size of 13 participants per group was needed. For VAS the minimum total sample was 39 (alpha = 0.05, power = 0.99, effect size = 1) [17]. For KOOS the minimum total sample was 45 (alpha = 0.05, power = 0.8, effect size = 0.81) [18]. Accounting for potential dropouts, the total sample size was increased to 60, with 20 participants assigned to each group.

### Randomization and allocation

Participants were randomly allocated to 3 groups with a ratio of 1:1:1. The computer-generated randomization method (Random Function; Microsoft Excel, MS Office 365) assigned patients to a specific treatment modality group. An independent statistician prepared the computer-generated randomization sheet. The schedule was hidden in opaque, sealed envelopes that were consecutively numbered. The envelopes were kept in a locker and opened sequentially for each stratum to disclose the group assignment. After the initial screening, the principal investigator received a treatment assignment from a physiotherapist working in SIPMR. Participants were blinded to the group allocation concerning the potential effect of the individual treatment protocol, and a different time slot was allotted for each intervention used as a strategy to reduce the interaction between the participants. The assessor, blind to group allocation, recorded outcomes at baseline after the third and sixth weeks.

### Intervention

Group 1 (Mulligan Mobilization with Movement, MWM) received Mulligan joint mobilization, knee strengthening, and kinesiotaping. Group 2 (Trunk Stabilization Exercises, TSE) underwent trunk stabilization exercises, kinesiotaping, and knee strengthening. Group 3 received kinesiotaping and knee strengthening. We used TiDieR checklist to report the findings of the intervention [19]. Each group underwent a 6-week intervention comprising 24 sessions (four sessions per week, each lasting 40 min). Table 1.

**Table 1:** Summary of Intervention Components, Exercises, and Prescription Details for Each Study Group

Group	Intervention Components	Exercises	Sets	Repetitions	Frequency	Total Sessions
Group 1	Mulligan Mobilization with Movement	MWM (Transverse, Sagittal, Frontal Directions)	3	6–10	4 sessions/week	24
		Knee Strengthening (Isometric Quadriceps)	3	10	4 sessions/week	24
		Straight leg raise exercise	3	10	4 sessions/week	24
		Kinesiotaping	-	-		
Group 2	Trunk Stabilization Exercises	Prone Extension of Both Lower Limbs	3	6–8	4 sessions/week	24
		Back Bridge	3	6–8	4 sessions/week	24
		Unilateral Back Bridge	3	6–8	4 sessions/week	24
		Sideways Step Up	3	6–8	4 sessions/week	24
		Knee Strengthening (Isometric Quadriceps)	3	10	4 sessions/week	24
		Straight leg raise exercise	3	10	4 sessions/week	24
		Kinesiotaping	-	-		
Group 3	Knee Strengthening	Straight Leg Raise	3	10	4 sessions/week	24
		Isometric Quadriceps Exercise	3	10	4 sessions/week	24
		Kinesiotaping	-	-		

### Mulligan Joint Mobilization (MWM)

Participants underwent sustained manual glides in multiple directions while supine. Frontal plane glides were tested first, followed by sagittal plane glides, and then rotation. The glide direction that reduced pain was used for MWM, with 6–10 repetitions across three sets in various planes, progressing from non-weight-bearing to weight-bearing as tolerated [20]. 

### Trunk Stabilization Exercises (TSE)

Trunk stabilization exercises consisted of four exercises: extension of both lower limbs in prone lying, back bridge, unilateral back bridge, and sideways-step up. Six to eight repetitions with three sets were performed in each session, followed by a 30-second rest between sets [15].

**Procedural detail**:

i. Prone cross-extension of the lower limbs, followed by lowering without bending the knees, stabilizing the lumbar area with a belt [21].
ii. Back bridge

Participants lifted their spine, thighs, and pelvis while supine, with thier knees bent at 90 degrees.

iii. Unilateral back bridge

Extension of one leg from the back-bridge position.

iv. Sideways step-up

Stepping up sideways on a 10 cm high stepper.

### Knee strengthening exercises

The two isometric strengthening exercises were the straight leg raise and the isometric quadriceps exercise. The isometric strengthening exercise consisted of 10 reps per set at the start of the intervention. It was gradually increased to two sets in the third week, followed by three sets until the end of the intervention. [22, 23].

**Procedural detail**:

i. **Isometric quadriceps maximum exercise**:

The participant had to lie supine on the bed with a rolled pillow under the subject’s knee. Patients were instructed to ensure quadriceps activation for 5 s while pressing the knee downward.

ii. **Straight leg raising (SLR) exercise**:

The patient was supine with the opposite leg bent for support. They were instructed to squeeze the quadricep muscles to their maximum before lifting the foot off the floor. They were further taught to slowly lift the leg 4 inches above the plinth and hold this position for 10 s [22, 23].

### Kinesiotaping

The muscle deloading taping used one Y and two I straps. The Y-tape base was affixed over the top of the patella in the maximum-stretched position of the knee, and then both ends of the Y-tape were secured around the knee joint, ending on the tibial tubercle. One I strap was applied at the level of the medial collateral ligament. Another one was applied over the lateral collateral ligament. The tape was changed after every session [24]. 

### Outcome measures

#### Primary outcomes

##### Knee Injury and Osteoarthritis Outcome score

The primary outcome was the knee injury and Osteoarthritis Outcome Score (KOOS), a validated 42-item patient-centered questionnaire assessing pain, symptoms, daily activities, sports activities, and quality of life in the Urdu language [25]. A score of 0 (extreme problems) or 100 (smoothly) was obtained separately for each sub-range. Minimally clinical important difference (MCID) was 15.4 for KOOS pain, 15.1 for KOOS symptoms, 17 for KOOS ADL, 11.2 for KOOS sports/recreation, and 16.5 for KOOS quality of life [26]. 

### Secondary outcomes

#### Visual Analogue Scale

VAS is a reliable unidimensional scale to assess pain severity that is used in various rheumatic diseases, including osteoarthritis [27]. The scale categorizes pain intensity as follows: no pain (0–4 mm), mild pain (5–44 mm), moderate pain (45–74 mm), and severe pain (75–100 mm). In our study, participants’ VAS readings were recorded during periods of rest and while ascending and descending stairs. These readings, reflecting the individuals’ pain experiences, were documented on a piece of paper for accurate assessment. MCID of pain was ranged from 0.84 to 0.9 [24, 25].

### 6-Minute Walk Test

The 6-Minute Walk Test (6MWT) is a sub-maximal exercise test involving the measurement of the distance walked over a period of six minutes [28]. Bright-colored tapes were utilized to mark each end of the 30-meter walkway. The environment was kept hazard-free, and readings were recorded by an assessor who had no knowledge of the group to which they belonged. The patients were instructed to wear comfortable shoes. MCID for 6-MWT ranged from 26 to 55 m [29].

### Stair climb test

The Stair Climb Test is used to measure the overall time taken by the participant to ascend and descend 11 stairs, each with a step height of 16 cm [28]. In cases where safety is a concern, the assessor walks behind the participant during the ascent and at the side during the descent. Alternatively, if safety is not a concern, the tester remains stationed at the start/finish position on the ground landing. MCID for SCT was 2.33 [29].

### Statistical analysis

Data were stored and analysed using SPSS version 23. The mean and standard deviation were presented for quantitative variables. Frequency and percentage were shown for categorical variables by applying the chi-square test. The Shapiro-Wilk test assessed normality. A mixed-design ANOVA (repeated measures with a between-subject factor) was used in this study to compare score changes measuring improvement in outcome measures. When significant main effects were found, the Bonferroni test was used to identify statistical differences. A value of < 0.05 was considered significant. In this research, the intention-to-treat approach was used. Missing data were imputed using the last observation carried forward, following CONSORT recommended guidelines.

## Results

**Fig. 1:**
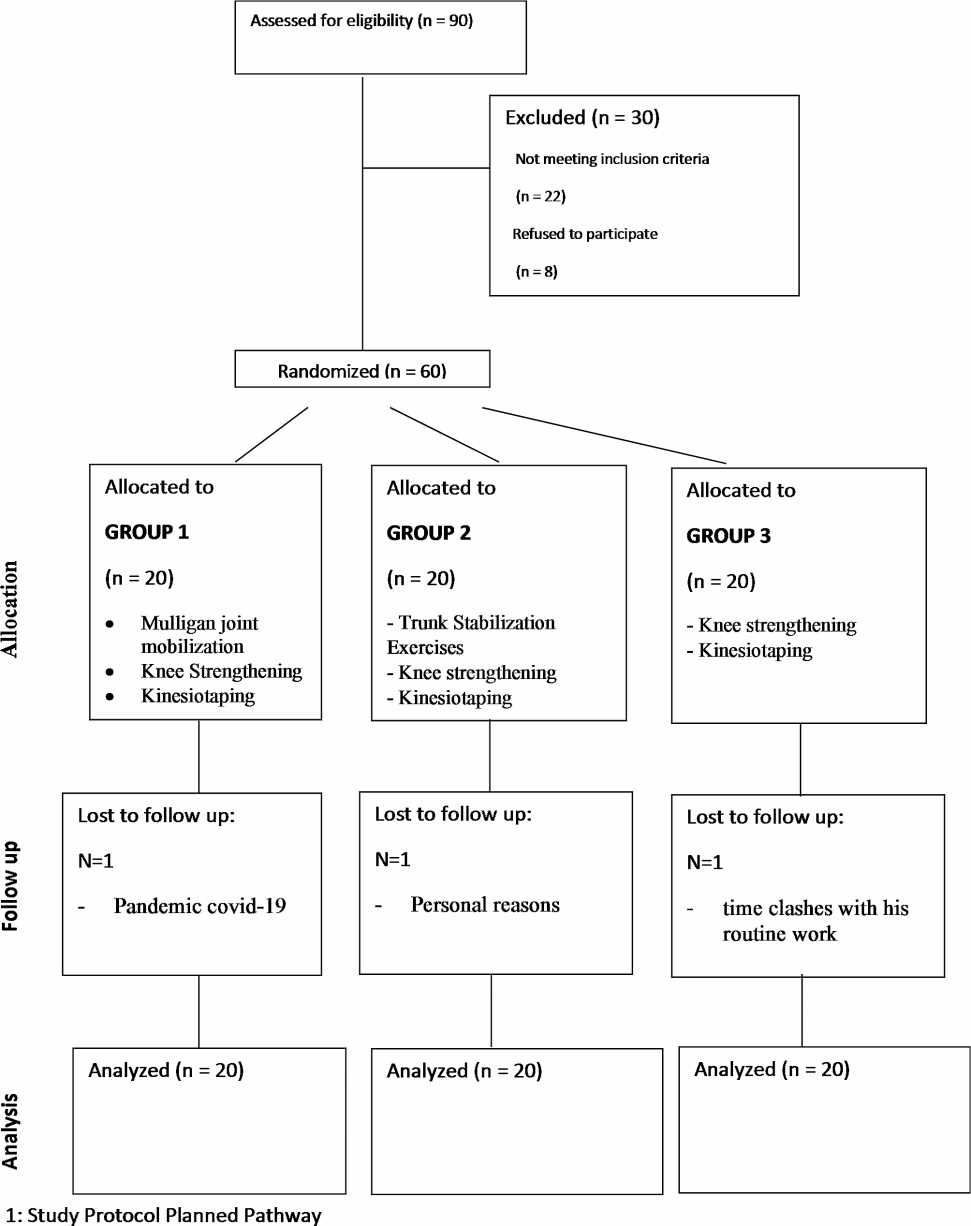
CONSORT diagram

Ninety KOA patients were screened for inclusion in the study. Sixty-eight fulfilled the criteria, and 60 participants agreed to participate in the study. Three patients dropped out from the study after three weeks because they could not attend physical therapy sessions (Fig. 1). No adverse event was reported in this trial.

There were 60 patients with a mean age of 50.77 ± 5.72 years, including 22 females. Of these, 37 patients (61.7%) were diagnosed with unilateral KOA, while 23 patients (38.3%) were presented with bilateral KOA. There were no significant differences between groups at the baseline regarding the demographic variables mentioned in Table 2.

**Table 2:** Baseline demographics of groups

Demographics	Group 1	Group 2	Group 3
**Age (years)**	49.95 ± 6.51	50.68 ± 5.19	51.68 ± 5.54
**BMI (Kg/m** ^**2**^ **)**	24.57 ± 1.89	25.09 ± 1.99	24.99 ± 2.23
**Symptom Duration (years)**	2.81 ± 1.21	3.10 ± 1.19	2.73 ± 1.24
**Involvement site**			
Unilateral KOA	13(35.2%)	12(32.4%)	12(32.4%)
Bilateral KOA	7(30.4%)	8(34.8%)	8(34.8%)
**Gender**			
Male	13(34.2%)	13(34.2%)	12(31.6%)
Female	7(31.8%)	7(31.8%)	8(36.4)
**K-L Criteria**			
Grade 1	3(30%)	4(40%)	3(30%)
Grade 2	17(34%)	16(32%)	17(34%)

Knee osteoarthritis; BMI: Body Mass Index; K-L: Kellgren-Lawrence; K-L grade 1: doubtful joint space narrowing and possible osteophytic lipping; K-L grade 2: definite osteophytes and possible joint space narrowing

### Knee Injury and Osteoarthritis Outcome score

In this study, the level of function assessed on KOOS exhibited a significant time interaction (F = 9.46, *p* = 0.001 for KOOS-Pain score; F = 5.01, *p* = 0.001 for KOOS-Symptoms score; F = 7.63, *p* = 0.001 for KOOS-ADL score; F = 4.58, *p* = 0.001 for KOOS-sports score; F = 2.84, *p* = 0.027 for KOOS-Qol score) with better improvement in Trunk Stabilization exercises from baseline to the 24th week of intervention. In the third week, TSE Group showed a greater difference (*P* < 0.05) from baseline in KOOS-Pain and KOOS-ADL. TSE Group and MWM Group demonstrated a greater decrease in KOOS-Pain in the sixth week. Group 2 showed a greater decrease in KOOS-Symptoms and KOOS-ADL at the sixth week. Effect sizes ranged from 0.13 to 0.55 (Table 3).

**Table 3:** Primary Outcomes at baseline, third, and sixth weeks

Group	Baseline		Third week		Sixth week	Week 3 Change	(*P*-value) *	Week 6 Change	(*P*-value) *	Effect Size
	Mean (SD)		Mean (SD)		Mean (SD)	Within-group score change Mean [95% CI]		Within-group score change Mean [95% CI]		(Between group at 24th session) ***
				KOOS-Pain						
1	34.21 ± 7.40		59.05 ± 5.09		75.68 ± 8.33	24.84 (16.64, 33.04)	< 0.001*	41.47 (34.57, 48.37)	< 0.001*	0.55
2	39.31 ± 9.04		68.42 ± 9.81		88.63 ± 8.39	27.77 (20.90, 37.30)	< 0.001*	49.11 (42.41, 56.21)	< 0.001*	
3	37.36 ± 10.35		43 ± 15.5		66 ± 12.44	5.36 (-2.56, 13.83)	0.286	28.73 (21.83, 35.63)	< 0.001*	
P-value**						0.001^b^		0.000^a, b^		
				**KOOS-Symptoms**						
1	27.94 ± 6.21		55.78 ± 8.43		75.15 ± 11.31	27.84 (20.46, 35.22)	< 0.001*	47.21 (40.66, 53.75)	< 0.001*	0.185
2	27.31 ± 10.72		59.47 ± 10		81.89 ± 6.46	32.15 (24.77, 39.53)	< 0.001*	54.57 (48.03, 61.12)	< 0.001*	
3	31.05 ± 4.10		50.15 ± 13.28		66.36 ± 12.91	19.1 (11.72, 26.48)	< 0.001*	35.31 (28.77, 41.86)	< 0.001*	
P-value**						0.06		0.000^b^		
				**KOOS-Activities of Daily Living**						
1	34.57 ± 8.55		55.42 ± 8.16		74.89 ± 11.58	20.84 (12.32, 29.35)	< 0.001*	40.31 (33.21, 47.42)	< 0.001*	0.29
2	33.10 ± 7.47		61.94 ± 10.08		83.68 ± 5	28.84 (20.32, 37.35)	< 0.001*	50.57 (43.47, 57.68)	< 0.001*	
3	36.63 ± 7.77		49.31 ± 19.20		62.68 ± 9.26	12.68 (4.16, 21.2)	< 0.001*	26.05 (18.94, 33.15)	< 0.001*	
P-value**						0.020^b^		0.000^b^		
				**KOOS-Sport and Recreation function**						
1	30.52 ± 13.63		61.31 ± 12		82.1 ± 14.84	30.78 (20.38, 39.19)	< 0.001*	51.57 (42.96, 58.19)	< 0.001*	0.148
2	34.21 ± 12.04		66.84 ± 9.88		85 ± 8.20	31.11 (24.22, 41)	< 0.001*	51.11 (43.96, 59.19)	< 0.001*	
3	37.89 ± 9.76		51.35 ± 24.99		77.89 ± 4.18	15.75 (5, 21.83)	0.08	41 (32.83, 47.61)	< 0.001*	
P-value**						0.051		0.063		
				**KOOS-Quality of Life**						
1	47.10 ± 5.98		70.78 ± 8.79		81.05 ± 14.42	23.68 (15.5, 31.86)	< 0.001*	33.94 (26.35, 41.54)	< 0.001*	0.134
2	47.3 ± 9.94		75.68 ± 9.95		93.15 ± 9.06	28.63 (20.44, 36.81)	< 0.001*	46.1 (38.5, 53.7)	< 0.001*	
3	47.09 ± 9.98		66.84 ± 9.88		89.84 ± 10.89	19.78 (11.60, 27.97)	< 0.001*	42.78 (35.19, 50.38)	< 0.001*	
P-value**						0.498		0.068		

Group 1: Mulligan mobilization with movement; Group 2: Trunk Stabilization Exercises; Group 3: Knee strengthening exercise; a: Statistically significant difference between Group 1 and 3; b: Statistically significant difference between Group 2 and 3; *statistically significant within-group differences; **Statistically significant between-group differences; ***Between-group (MwM vs. TSE vs. KSE) effect sizes at 24 sessions.

### Visual Analogue Scale

The within-group analysis revealed significant differences in VAS measurements, both at rest and during stair ascent and descent, across the three assessment phases. The effect sizes, ranging from 0.34 to 0.44, are detailed in Table 4.

#### Visual Analogue Scale at Rest

A significant interaction between time and group was observed for VAS at rest (F = 3.42, *p* = 0.001). This indicates a statistically significant difference in pain reduction at rest among the groups. Analysis of VAS values in the third week demonstrated a greater reduction in pain for the TSE and MWM groups. In the sixth week, only TSE Group exhibited a greater reduction in rest-related pain (Table 4).

#### Visual analogue scale score during stair ascent and descent

When assessing pain during stair ascent, a significant interaction between time and group was found (F = 6.81, *p* = 0.001 for VAS-Ascending stairs), with both TSE Group and MWM Group showing increased improvement in the third and sixth weeks. Similarly, during stair descent, a significant interaction was observed (F = 6.27, *p* = 0.001 for VAS-Descending stairs). TSE Group and MWM Group demonstrated a significant difference in the third week, and in the sixth week, the improvement in pain during descent was more in TSE Group (Table 4).

#### 6-Minute Walk Test (6MWT)

The 6MWT also demonstrated a significant interaction between time and group (F = 28.73, *p* = 0.001). There was a greater mean difference in TSE group and MWM group in the third week, and after the 6-week follow-up, an even greater difference was observed in the TSE and MWM groups (Table 4).

### Stair climb test (SCT)

SCT showed a significant interaction between time and group (F = 7.16, *p* = 0.001). However, the mean scores of all groups improved over time, with greater improvement seen in TSE Group and MWM Group.

**Table 4:** Secondary Outcomes at baseline, third, and sixth weeks

Group	Baseline		Third week		Sixth week	Week 3 Change	(*P*-value) *	Week 6 Change	(*P*-value) *	Effect Size
	Mean (SD)		Mean (SD)		Mean (SD)	Within-group score change Mean [95% CI]		Within-group score change Mean [95% CI]		(Between group at 24th session) ***
				Visual Analogue scale recorded at rest						
1	6.15 ± 0.95		4.10 ± 0.8		2 ± 0.88	-2.05 (-2.78, -1.32)	< 0.001*	-4.15 (-4.80, -3.40)	< 0.001*	0.34
2	5.78 ± 1.13		3.42 ± 0.69		1.36 ± 0.49	-2.38(-3.10, -1.63)	< 0.001*	-4.42 (-5.12, -3.71)	< 0.001*	
3	6.14 ± 0.93		4.8 ± 1.04		2.36 ± 1.06	-1.05 (-1.78, -0.32)	< 0.001*	-3.78 (-4.49, -3.08)	< 0.001*	
P-value**						0.002^a, b^		0.001^b^		
				**Visual Analogue scale recorded at ascending stairs**						
1	5.73 ± 1.19		3.78 ± 0.71		1.84 ± 0.95	-1.94 (-2.65, -1.24)	< 0.001*	-3.89 (-4.74, -3.04)	< 0.001*	0.448
2	6.31 ± 1.05		3.81 ± 0.73		1.42 ± 0.5	-2.5 (-3.28, -1.87)	< 0.001*	-4.88 (-5.74, -4.40)	< 0.001*	
3	6.26 ± 1.24		5.63 ± 0.59		2.68 ± 1.00	-0.63 (-1.33, 0.09)	0.095	-3.56 (-4.43, -2.72)	< 0.001*	
P-value**						0.000^,a, b^		0.004^,a, b^		
				**Visual Analogue scale recorded at descending stairs**						
1	5.73 ± 1.147		4.31 ± 0.67		1.94 ± 0.91	-1.42 (-2.10, -0.74)	< 0.001*	-3.78 (-4.58, -3)	< 0.001*	0.351
2	5.57 ± 0.76		3.26 ± 0.45		1.52 ± 0.51	-2.33 (-3, -1.63	< 0.001*	-4.05 (-4.84, -3.26)	< 0.001*	
3	5.42 ± 1.01		4.94 ± 0.70		2.47 ± 0.96	-0.47 (-1.15, 0.2)	0.271	-2.94 (-3.73, -2.15)	< 0.001*	
P-value**						0.001^a, b^		0.003^b^		
				**6-Minute walk test**						
1	352.47 ± 18.80		365.36 ± 18.83		374.89 ± 18.93	12.89 (17.07, 8.71)	< 0.001*	22.42 (17.68, 27.16)	< 0.001*	0.16
2	344.57 ± 36.72		367.25 ± 34.42		382.21 ± 33.12	22.68 (18.5, 28.68)	< 0.001*	37.6 (32.89, 42.37)	< 0.001*	
3	332.73 ± 25.05		340.63 ± 24.59		346.57 ± 25.50	7.89 (3.71, 12.07)	< 0.001*	13.84 (9.10, 18.58)	< 0.001*	
P-value**						0.005^a, b^		0.001^,a, b^		
				**11-Stair Climb test**						
1	18.84 ± 1.89		15.05 ± 2.34		11.89 ± 1.69	-3.89 (-4.93, -2.86)	< 0.001*	-7.05 (-8.18, -5.91)	< 0.001*	0.14
2	18.94 ± 2.41		14.68 ± 1.79		12 ± 1.29	-4.26 (-5.29, -3.22)	< 0.001*	-7.16 (-8.08, -5.81)	< 0.001*	
3	18.42 ± 1.26		16.57 ± 1.12		14.21 ± 1.84	-1.84 (-2.87, -0.80)	< 0.001*	-4.21 (-5.3, -3.07)	< 0.001*	
P-value**						0.005^a, b^		0.000 ^a, b^		

Group 1: Mulligan mobilization with movement; Group 2: Trunk Stabilization Exercises; Group 3: Knee strengthening exercise; a: Statistically significant difference between Group 1 and 3; b: Statistically significant difference between Group 2 and 3; *statistically significant within-group differences; **Statistically significant between-group differences; ***Between-group (MwM vs. TSE vs. KSE) effect sizes at 24 sessions.

## Discussion

The study aimed to evaluate the effects of trunk stabilization exercises (TSE) and Mulligan joint mobilization (MWM) on pain, disability, and sub-maximal exercise capacity in patients with KOA compared with isometric knee strengthening. The findings suggested that trunk stabilization exercises were more effective in alleviating pain intensity, reducing disability, and improving walking capacity among KOA patients than Mulligan joint mobilization and isometric knee strengthening exercises.

TSE and MWM resulted in more pain reduction at rest, as measured by VAS. The mean difference in VAS scores in TSE was − 2.38 cm and − 4.42 cm in the third week and the sixth week, respectively, more than a minimal clinically relevant difference of 0.84 cm [27]. Similarly, the intensity of pain reduction in the current research was greater than in a previous study conducted on trunk stability exercises. It was probably because the previous study had more females, a higher BMI, NSAID use, and walking aid reliance [15]. A comparative study on MWM combined with other treatments resulted in similar effects [31, 32].

During stair ascent, both MWM and TSE groups experienced clinically significant pain relief, with an average reduction of 1.9 cm in VAS, which is more than the minimal clinically important difference [27]. But it should be noted that the within-group change scores for pain at both post-intervention times were greater than 2.0 cm only for the TSE group. This improvement may be attributed to various factors, for instance, reduced onset muscle activity detected in the stair-stepping task using EMG, reduced onset activity of the posterior portion of the gluteus medius (GM), leading to decreased activity of the tensor fascia lata, neuromuscular control of the GM, and trunk side flexion strength [33]. TSE, assessed with EMG studies, demonstrates that TSE is a more useful treatment method [30, 34–36]. 

The compensatory mechanism of stair descending in KOA patients has been documented, but its impact on pain after muscle strengthening is less explored [37, 38]. The TSE group in this study showed a reduction in pain during stair descent in the 6th week, likely due to altered muscle activation patterns, particularly in the vastus medialis oblique, vastus lateralis, and gluteus medius. This suggests that muscle strengthening interventions may influence pain perception during stair descent by modifying muscle activation patterns [39]. 

The 6-Minute Walk Test (6MWT), a reliable and accurate measure recommended by the Osteoarthritis Research Society International (OARSI), showed improved exercise capacity in both MWM and TSE groups [29]. In the current study, sub-maximal exercise capacity improved in the third and sixth weeks in the MWM and TSE groups. Dobson et al. suggested that 6MWT has a minimal detectable change (MDC) of 7.6% and a standard error of measurement (SEM) of 3.3% [29]. The 6-MWT reading of the average change score exceeded the MDC values (i.e., the within-group mean difference on 6-MWT was MWM 22.42 and TSE 37.6). This contradicts earlier studies that reported a minimal impact of trunk stabilization exercises on sub-maximal exercise capacity, possibly due to variations in participant age, higher BMI, and sample size (more females) [15]. Kulkarni et al. conducted a study on the effects of MWM with and without conventional treatment in the management of KOA and demonstrated no significant difference in the 6MWT score between both groups. This may be attributed to differences in treatment sessions [40]. 

SCT is another measure recommended by OARSI to assess the time taken to ascend and descend 11 steps with a step height of 16 cm [29]. In ascending stairs, participants with KOA demonstrated a slower ascent and increased trunk motion in both limbs [41]. During descent, there was a reduction in trunk transverse motion in both limbs, increased knee frontal motion in the affected limb, and modified trunk sagittal and knee transverse motions in the unaffected limbs [41]. This underscores the importance of trunk muscle strength in addressing functional limitations, a notion supported by significant improvements in SCT performance in the TSE group [11–14]. SCT has an MDC of 2.23 and a SEM of 1.00.^29^ The SCT reading of the average change score exceeded the MDC values (i.e., within-group mean difference on TSE 7.30). The result of the study reveals significant improvement in SCT at the end of the trial in the TS group, which comprises 24 sessions of supervised exercise. Previous studies opted for 8 and 24 sessions of intervention while using the SCT test [42, 43]. However, the study based on exercise dosing showed that those studies, which had fewer than 24 sessions, may impact to replicate the similar effects [7]. 

In evaluating the Knee Injury and Osteoarthritis Outcome Score (KOOS) for pain, the TSE group showed marked improvement by the third week, outperforming other groups. Upon reassessment at the sixth week, both the TSE and MWM groups demonstrated significant improvement in the KOOS-Pain score. These findings contrast with previous studies on treatment effects due to variations in participant demographics, such as a higher proportion of females, an older mean age, and incomplete information regarding the chronicity of KOA and the affected knee compartment [15]. Conversely, an RCT found no significant difference in KOOS scores between the MWM and Muscle Energy Technique [44]. This might be due to the smaller sample size and differences in treatment sessions. It is hypothesized that postural instability in patients with moderate-to-severe medial KOA is correlated with diminished ability to perform daily activities (ADL) and reduced quality of life (QOL) [45]. Postural instability can increase the risk of falls and subsequent injuries, further impacting the patient’s overall well-being. Therefore, it is crucial for individuals with medial KOA to receive appropriate treatment aimed at improving postural stability and reducing the likelihood of falls and injuries. TSE can be utilized to enhance postural control.

KOA and hip OA result in decreased muscle strength and atrophy of the muscles surrounding the knee joint [46]. Knee strengthening alone seems less effective in improving function and pain in patients with KOA. Therefore, multimodal exercises for the knee are recommended for better pain relief than isometric knee strengthening [42, 43]. TSE improves stability and coordination of the knee, hip, and pelvis by activating key periarticular muscles of the knee and lumbopelvic-hip complex. There is evidence that exercises targeted at strengthening the muscles proximal to the knee are beneficial for reducing knee pain [43, 48–50].

MWM works by correcting the positional fault by restoring the joint arthrokinematics [8, 9]. Joint mobilization combined with taping and strengthening exercises helps to maintain the correcting effects of mobilization in walking and pain during climbing and ascending stairs [46]. This improvement is thought to result from biomechanical factors. For example, forces acting on the knee joint vary as the position of the body changes from extension to flexion. Since walking down and up the stairs in alternative steps causes both loadings in flexion and load transfer on a single leg, more loads are imposed on the knee [8, 13]. Similar to the results of the current study, a previous study also reported that MWM, in combination with taping, provides pain relief and improves function. However, this was seen only in female participants [10]. Our study included both genders.

For our study, we only selected tibiofemoral joints based on radiological and clinical criteria. In cases of bilateral KOA, the more symptomatic knee was chosen for outcome measurement. No patients reported in this trial reported increased pain during or after the exercise. Participants tending to avoid activity due to pain in the initial sessions of intervention might explain possible reasons to achieve improvement. After the symptoms were resolved, participants were motivated to perform the exercise. As a result, no adverse events were reported in this trial.

There are some limitations that warrant mention. Firstly, as a single-center study with a modest sample size, the generalizability of our findings may be restricted. Future research endeavors should meticulously consider both sample size and effect size, particularly in the context of prolonged exercise interventions aimed at evaluating pain and functional disability, as these factors may yield diverse outcomes. Moreover, the exclusion of individuals with higher BMI and concurrent low back pain could potentially limit the applicability of our findings to broader populations. Therefore, there is a need for longer-term interventions and studies involving larger cohorts to further investigate the efficacy of these treatments in advanced knee osteoarthritis (KOA) and to monitor physical activity levels across extended follow-up periods.

## Conclusion

As compared to Mulligan joint mobilization and isometric knee strengthening exercises, trunk stabilization exercises were more beneficial in alleviating pain intensity, reducing disability, and improving walking capacity in a selected set of patients with KOA. Positive outcomes in daily activities and functional tests emphasize the practical benefits of these interventions, offering valuable insights for clinicians aiming to enhance the well-being of KOA patients.

### Electronic supplementary material

Below is the link to the electronic supplementary material.

Supplementary Material 1

Supplementary Material 2

## Data Availability

Data included in the current study are not publicly available to ensure confidentiality of the patients but are available from the corresponding author on reasonable request.

## Abbreviations

6-MWT: 6-minute walk test
ACR: American College of Rheumatology
DUHS: Dow University of Health Sciences
GM: Gluteus Medius
IRB: Institutional review board
K/L: Kellgren Lawrence
Knee OA: Knee Osteoarthritis
KOOS: Knee injury and Osteoarthritis Outcome Score
MT: Manual therapy
MCD: Minimally detectable Change
MWM: mobilization with movement
OA: Osteoarthritis
OARSI: Osteoarthritis Research Society International
OPD: Outpatient department
PI: Principal investigator
RCT: Randomized controlled trial
SEM: standard error of measurement
SCT: Stair Climb Test
SIPM&R: Institute of Physical Medicine & Rehabilitation, Health Department, Government of Sindh
TSE: Trunk stabilization exercises
VAS: Visual Analogue Scale
VAS-ASCENDSTAIR: Visual Analogue Scale at ascending stairs
VAS-DESCENDSTAIR: Visual Analogue Scale at descending stairs
