# Supplementary material for: Efficacy of Mulligan joint mobilizations and trunk stabilization exercises versus isometric knee strengthening in the management of knee osteoarthritis: a randomized controlled trial
Source: BMC Sports Sci Med Rehabil. 2024 May 7;16:105. doi: 10.1186/s13102-024-00893-7 (PMC11075249; doi:10.1186/s13102-024-00893-7)
Supplement: Supplementary file 1 — Supplementary Material 1 [file 13102_2024_893_MOESM1_ESM.docx]

| **S.no** | **Group 1** | **Group 2** | **Group 3** |
| --- | --- | --- | --- |
| 1 | Mulligan Mobilization with movement | Trunk Stabilization Exercises | Knee strengthening exercises |
| 2 | Knee strengthening exercises | Knee strengthening exercises | Kinesiotaping |
| 3 | Kinesiotaping | Kinesiotaping |  |

**Procedural details for the intervention in this study**

**Knee Strengthening Exercise**


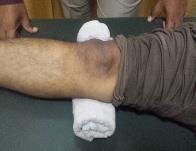

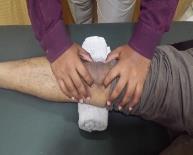


**Isometric quadriceps Initial Position Isometric Quadriceps Final Position**


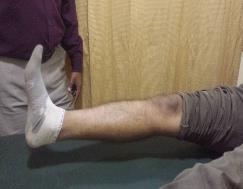


**Isometric Straight leg raise**

**Mulligan Mobilization with movement**


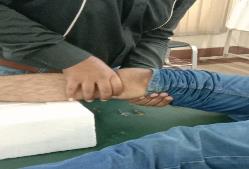

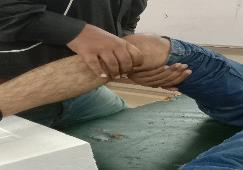


**Anteroposterior GLIDE**


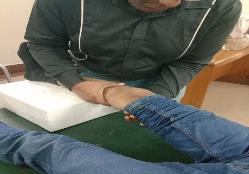


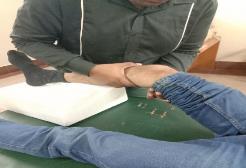


**Posterioanterior GLIDE**


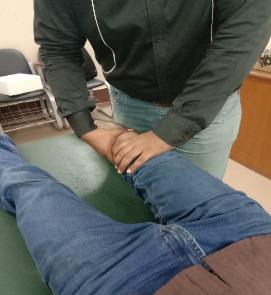

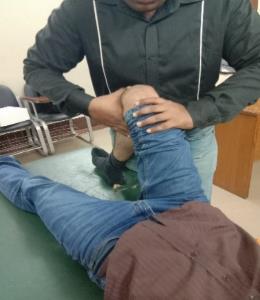


**LATERAL GLIDE in flexion and extension**


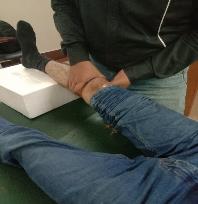


**MEDIAL GLIDE**


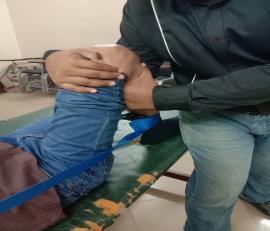


**External rotation in flexion and extension**


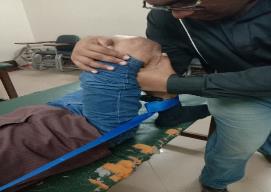


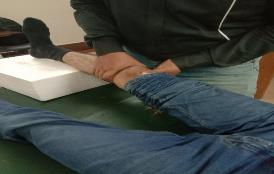


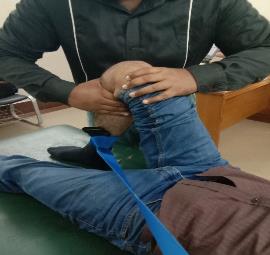


**Internal Rotation in flexion and extension**


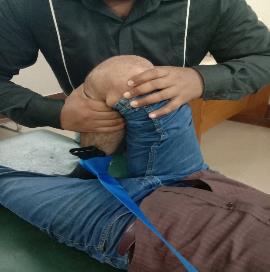


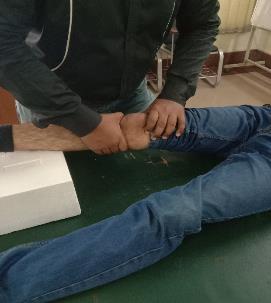


**Kinesiotaping**


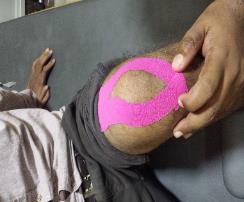

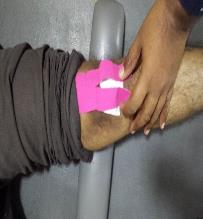


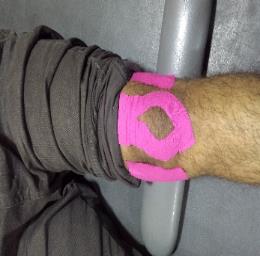


**Initial position Knee flexion Position**

**Final position**

**Trunk Stabilization Exercises**


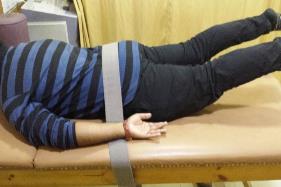


**Trunk stabilization exercise using the belt.**


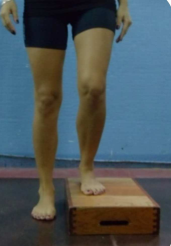

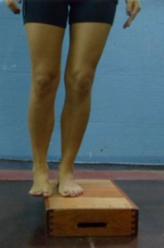


**Lateral step up**


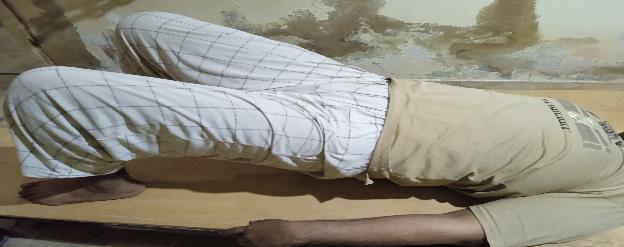


Back Bridge


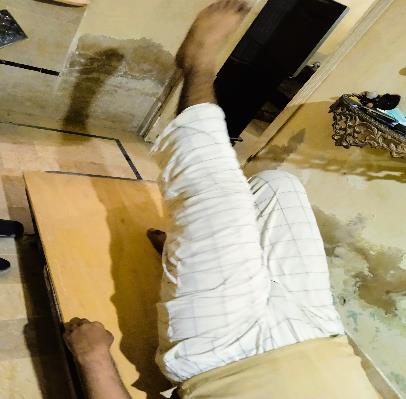


Unilateral Back Bridge
