# Supplementary material for: Efficacy of Mulligan joint mobilizations and trunk stabilization exercises versus isometric knee strengthening in the management of knee osteoarthritis: a randomized controlled trial
Source: BMC Sports Sci Med Rehabil. 2024 May 7;16:105. doi: 10.1186/s13102-024-00893-7 (PMC11075249; doi:10.1186/s13102-024-00893-7)
Supplement: Supplementary file 2 — Supplementary Material 2 [file 13102_2024_893_MOESM2_ESM.docx]

The TIDieR (Template for Intervention Description and Replication) Checklist*:


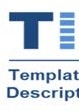

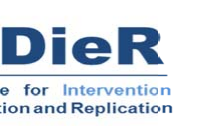


Information to include when describing an inter ention and the location of the information

m

| **Item Item Where located **** | |
| --- | --- |
| **number** Pri ary paper  (page or appendix number) | Other ^†^ (details) |
| **BRIEF NAME 7-10**   1. Provide the name or a phrase that describes the intervention.   **WHY**   1. Describe any rationale, theory, or goal of the elements essential to the intervention. 4-5   **WHAT**   1. Materials: Describe any physical or informational materials used in the intervention, including those 7-10 provided to participants or used in intervention delivery or in training of intervention providers.   Provide information on where the materials can be accessed (e.g. online appendix, URL).   1. Procedures: Describe each of the procedures, activities, and/or processes used in the intervention, 7-10 including any enabling or support activities.   **WHO PROVIDED**   1. For each category of intervention provider (e.g. psychologist, nursing assistant), describe their 7 expertise, background and any specific training given.   **HOW**   1. Describe the modes of delivery (e.g. face-to-face or by some other mechanism, such as internet or 7-10 telephone) of the intervention and whether it was provided individually or in a group.   **WHERE**   1. Describe the type(s) of location(s) where the intervention occurred, including any necessary 6 infrastructure or relevant features. | SUPERVISED EXERCISE SESSION |

| **WHEN and HOW MUCH**   1. Describe the number of times the intervention was delivered and over what period of time including 8 the number of sessions, their schedule, and their duration, intensity or dose.   **TAILORING**   1. If the intervention was planned to be personalised, titrated or adapted, then describe what, why, - when, and how.   **MODIFICATIONS**   1. **^ǂ^** If the intervention was modified during the course of the study, describe the changes (what, why, - when, and how).   **HOW WELL**   1. Planned: If intervention adherence or fidelity was assessed, describe how and by whom, and if any - strategies were used to maintain or improve fidelity, describe them. 2. **^ǂ^** Actual: If intervention adherence or fidelity was assessed, describe the extent to which the -   intervention was delivered as planned. |  |
| --- | --- |

** **Authors** - use N/A if an item is not applicable for the intervention being described. **Reviewers** – use ‘?’ if information about the element is not reported/not sufficiently reported.

† If the information is not provided in the primary paper, give details of where this information is available. This may include locations such as a published protocol or other published papers (provide citation details) or a website (provide the URL).

ǂ If completing the TIDieR checklist for a protocol, these items are not relevant to the protocol and cannot be described until the study is complete.

- We strongly recommend using this checklist in conjunction with the TIDieR guide (see *BMJ* 2014;348:g1687) which contains an explanation and elaboration for each item.
- The focus of TIDieR is on reporting details of the intervention elements (and where relevant, comparison elements) of a study. Other elements and methodological features of studies are covered by other reporting statements and checklists and have not been duplicated as part of the TIDieR checklist. When a **randomised trial** is being reported, the TIDieR checklist should be used in conjunction with the CONSORT statement (see www.consort‐statement.org) as an extension of **Item 5 of the CONSORT 2010 Statement.**

When a **clinical trial protocol** is being reported, the TIDieR checklist should be used in conjunction with the SPIRIT statement as an extension of **Item 11 of the SPIRIT 2013 Statement** (see www.spirit‐statement.org). For alternate study designs, TIDieR can be used in conjunction with the appropriate checklist for that study design (see www.equator‐network.org).
